# Developing a Computational Phenotype of the Fourth Universal Definition of Myocardial Infarction for Inpatients

**DOI:** 10.3390/jcm13247773

**Published:** 2024-12-19

**Authors:** Elliot A. Martin, Bryan Har, Robin L. Walker, Danielle A. Southern, Hude Quan, Cathy A. Eastwood

**Affiliations:** 1 Centre for Health Informatics, Cumming School of Medicine, University of Calgary, Calgary, AB T2N 4Z6, Canada; eamartin@ucalgary.ca (E.A.M.); robin.walker@ucalgary.ca (R.L.W.); dasouthe@ucalgary.ca (D.A.S.); caeastwo@ucalgary.ca (C.A.E.); 2 Provincial Research Data Services, Alberta Health Services, Calgary, AB T2N 4Z6, Canada; 3 Alberta Health Services, Calgary, AB T3B 0N9, Canada; bjnhar@ucalgary.ca; 4 Libin Cardiovascular Institute, Cumming School of Medicine, University of Calgary, Calgary, AB T2N 4N1, Canada; 5 Department of Community Health Science, Cumming School of Medicine, University of Calgary, Calgary, AB T2N 4Z6, Canada

**Keywords:** phenotype, myocardial infarction, electronic medical records

## Abstract

**Background**: The fourth universal definition of myocardial infarction (MI) introduced the differentiation of acute myocardial injury from MI. In this study, we developed a computational phenotype for distinct identification of acute myocardial injury and MI within electronic medical records (EMRs). **Methods**: Two cohorts were used from a Calgary-wide EMR system: a chart review of 3042 randomly selected inpatients from Dec 2014 to Jun 2015; and 11,685 episodes of care that included cardiac catheterization from Jan 2013 to Apr 2017. Electrocardiogram (ECG) reports were processed using natural language processing and combined with high-sensitivity troponin lab results to classify patients as having an acute myocardial injury, MI, or neither. **Results**: For patients with an MI diagnosis, only 64.0% (65.7%) in the catheterized cohorts (chart review cohort) had two troponin measurements within 6 h of each other. For patients with two troponin measurements within 6 h; of those with an MI diagnosis, our phenotype classified 25.2% (31.3%) with an acute myocardial injury and 62.2% (55.2%) with an MI in the catheterized cohort (chart review cohort); and of those without an MI diagnosis, our phenotype classified 12.9% (12.4%) with an acute myocardial injury and 10.0% (13.1%) with an MI in the catheterized cohort (chart review cohort). **Conclusions**: Patients with two troponin measurements within 6 h, identified by our phenotype as having either an acute myocardial injury or MI, will at least meet the diagnostic criteria for an acute myocardial injury (barring lab errors) and indicate many previously uncaptured cases. Myocardial infarctions are harder to be certain of because ECG report findings might be superseded by evidence not included in our phenotype, or due to errors with the natural language processing.

## 1. Introduction

In 2018, the fourth universal definition of myocardial infarction (MI) was introduced, representing the latest and most comprehensive worldwide definition [1]. Unlike the previous definitions, this definition incorporates a pivotal feature, the differentiation of acute myocardial injury from MI [1]. After the introduction of the third universal definition of myocardial infarction [2], multiple studies emerged showing people with a myocardial injury had an increased mortality risk [3,4,5,6]. Following the introduction of the fourth universal definition of myocardial infarction, evidence indicates that adjusted mortality risk is even higher for people with an acute myocardial injury than for a chronic one [7]. Throughout this paper, we consistently spell out ‘acute myocardial injury’, while abbreviating myocardial infarction as ‘MI’.

Electronic medical records (EMRs) are increasingly being adopted by healthcare systems. While EMR systems present numerous benefits for advancing scientific research and discovery, they also pose challenges in locating necessary data elements stored within large complex systems and the management of diverse data formats. Computational phenotyping serves as a methodology for translating this EMR data, often characterized by its complexity, into meaningful medical concepts.

By harnessing the potential of EMRs and leveraging computational phenotyping, we have the capacity to enhance the accuracy and efficiency of case identification, thus contributing to a more comprehensive understanding of cardiac conditions and their implications. Computational phenotypes can then play a vital role in supporting clinical decision-making processes and advancing health research within the broader framework of a learning health system. This synergy of EMRs and computational phenotyping promises to foster a more comprehensive understanding of cardiac health, facilitating informed medical decisions and advancing healthcare research.

In the context of the fourth universal definition of MI, the concept of computational phenotype was deliberated to move away from solely relying on administrative codes for case identification [1]. This shift in approach is crucial when considering the inherent limitations of coded administrative data, which may lack sensitivity and specificity. Therefore, this study aims to advance the development of a robust computational phenotype for MI. Specifically, the focus lies on identifying and differentiating cases involving acute myocardial injuries and MIs within EMR encounters.

## 2. Materials and Methods

### 2.1. Setting

Data were collected from patients with an emergency or inpatient encounter for the adult patient population hospitalized in the Calgary, Canada, area (data time frames described below in Section 2.2). Throughout this period, all Calgary hospitals utilized the same EMR system, Sunrise Clinical Manager [8], for tracking patient-level clinical information such as lab results and clinical documentation.

### 2.2. Data Sources

Two cohorts were used in this study. The first is a cardiac-specific cohort called CREATE [9] which was previously created for EMR phenotyping. The CREATE database includes EMR data linked to the Alberta Provincial Project for Outcome Assessment in Coronary Heart Disease (APPROACH) registry [10,11,12], the administrative Discharge Abstract Database (DAD) [13] and the National Ambulatory Care Reporting System (NACRS) [14]. The APPROACH registry captures all patients who undergo cardiac catheterization in the province of Alberta (both as inpatients or outpatients) and reliably captures disease status. Administrative data for admission at the time of catheterization was linked to capture full hospital admission episodes. The disease status labels captured within the APPROACH registry were then supplemented with administrative data codes. We restricted this cohort to encounters 2013–2017 as years prior to 2013 had a relative lack of high-sensitivity troponin labs. A total of 11,685 episodes of care were constructed from this dataset.

The second cohort was a chart review dataset [15] originally developed to compare the accuracy of International Classification of Diseases, 10th version, Canadian Coding Standard (ICD-10-CA) [16] with the World Health Organization’s newly developed ICD-11 system [17]. This chart review captured diagnoses of Charlson comorbidities [18], including MI status. A total of 3042 patients, aged 18 and older, were randomly selected from those with an inpatient encounter between December 2014 to June 2015. Patients were labeled “yes” when evidence of an MI was present, “no” when evidence was absent, and “maybe” when conflicting and/or incomplete information was present which prevented definitive classification after re-reviewing.

Both of these cohorts are from before the introduction of the 4th Universal Definition of MI, so do not include the classification of acute myocardial injury. However, we can compare how well our phenotype of the 4th Universal Definition aligns with historical MI diagnoses and assess whether acute myocardial injuries are being missed or misclassified into MI diagnoses.

### 2.3. Algorithm Development

In accordance with the fourth universal definition of MI, a patient must initially meet the criteria for acute myocardial injury before meeting additional conditions to be classified with an MI. These criteria are defined as an elevated cardiac troponin above the 99th percentile Upper Reference Level (URL), accompanied by rising or falling levels. Calgary labs use a Roche Elecsys high-sensitivity, fifth-generation, cardiac troponin T assay with a 99th percentile URL of 14 ng/L. Values in the EMR system are reported as integers when it is 3 ng/L or larger, and as ‘<3’ when smaller. To create the computational phenotype, we considered values of 14 ng/L or greater as elevated values and assigned a value of 2 to all instances marked as ‘<3’ for calculation purposes.

The fourth universal definition of MI recommends that troponins be measured at assessment and then again 3 to 6 h later, to assess a changing pattern. There are still differing opinions about how to quantify how large these changes must be to diagnose acute myocardial injury. An expert consensus group has recommended that an increase of 50% be used when the initial value is less than or equal to the URL, and a change of 20% be used when it is above the URL [19]. However, other studies have shown that absolute changes have higher diagnostic accuracy than relative ones [20,21]. For this reason, we first assessed the performance of the relative changes proposed in [19], as well as an absolute change of 10 ng/L proposed in [20] (where they found the optimal change for their high-sensitivity cardiac troponin T, with a URL of 14 ng/L, was ≥9.2 ng/L). We determined which threshold to use based on which would most accurately classify MI diagnoses in our two cohorts, by including as many MI diagnoses as possible and excluding as many negative cases as possible.

If a patient met the criteria for an acute myocardial injury, then we utilized ECG reports to see if they also met the criteria for MI. This was performed by finding evidence of current acute MI, infarction, or ischemia within the report. While other techniques can be used to detect MIs, such as echocardiography and cardiac magnetic resonance imaging, we restricted our phenotype to ECG reports as ECGs are much more commonly performed. Additionally, we did not analyze the raw ECG trace, but the clinical ECG reports that were confirmed by a physician and stored in the EMR. Free-text reports were processed using natural language processing. Analyzing free-text reports would normally be quite difficult given the complexities of language and medical documentation but was made easier as the reports are initially auto-populated by General Electric’s MUSE ECG system and can later be edited.

The ECG reports were processed using custom regular expressions [22], a powerful pattern-matching tool often used for NLP tasks. This is a rule-based system that is supported by EMR databases, which makes it easier to implement than machine learning models. The code we used can be found in [23]. We first discarded reports that showed no significant changes when compared to a previous ECG taken more than 1 day before the current encounter. The remaining ECG reports are preprocessed in 4 steps by removing text that might confound the analysis:

1. The header matter containing the reason for the test and vital signs were removed, only leaving the report body.
2. Text comparing the current ECG to previous encounters was removed, e.g., “When compared with ECG of 01-JAN-1901 24:59, No significant change was found”.
3. Findings that cited previous ECGs were removed, e.g., “Inferior infarct (cited on or before 01-JAN-1901)”.
4. Findings that are age undetermined were removed, e.g., “Inferior-posterior infarct, age undetermined”.

We then flagged ECG reports matching 3 regular expressions (ignoring capitalization) indicative of MI:

1. “\* acute mi”, which is automatically included by the MUSE system and surrounded by ‘*’s to draw attention to it.
2. “infarc”, which captures documentation of infarctions.
3. “ischem|ischaem”, which captures ischaemic, ischemia, etc.

Patients who met the criteria for acute myocardial injury and had an ECG report that contained any of these three patterns were subsequently classified as having an MI according to our phenotype. The Python code used for processing these ECG reports can be accessed in [23].

### 2.4. Statistical Analysis

We compared demographics for each cohort with proportions for categorical variables and mean (and standard deviation) for continuous variables.

Since this study involves a comparison of historical myocardial infarction documentation with the recently introduced criteria of the 4th Universal Definition of Myocardial Infarction, neither could be considered a gold standard. Therefore, we present our primary results in the form of simple matching coefficients [24] and contingency tables, illustrating the proportions that align with the historical labels, and those generated from our computational phenotype.

## 3. Results

### 3.1. Cohort Description

The CREATE cohort was older with more males than the chart review cohort (Table 1). Also, the CREATE cohort had a higher proportion of cardiac risk factors and comorbid conditions given it was a cardiac-specific cohort versus randomly selected hospital records.

In Table 2, we present the numbers and proportions of patients in each cohort who meet different troponin criteria for the designation of MI or acute myocardial injury, stratified by whether they had an MI diagnosis. It is important to note that while over 90% of patients with an MI diagnosis in both cohorts had at least two troponin labs, less than 70% of them had two within 6 h of each other. Since this would exclude more than 30% of previous MI diagnoses, we categorized our phenotype into two case types. The first involves patients with two troponin labs within 6 h of each other, and the second involves those who did not. Consequently, we present troponin changes within 6 h and over the entire encounter for both cohorts.

Table 2 also indicates that employing an absolute change threshold of 10 ng/L more closely matches previous diagnoses than the relative change thresholds, as it is present in both a higher proportion of cases with an MI diagnosis, and a lower proportion of cases without an MI diagnosis. The only exception occurs in the chart review cohort, where two troponin labs were available within 6 h. In this scenario, 2.2% of patients without an MI diagnosis exhibited a change of 10 ng/L, whereas 1.9% met the relative change threshold.

Table 3 shows a similar report to Table 2, except focusing on the ECG report criteria for the designation of MI or acute myocardial injury. Almost all patients with an MI diagnosis in either cohort have an ECG report. In the CREATE cohort an almost equal proportion without an MI diagnosis also had an ECG report; however, that dropped to 56.5% in the chart review cohort.

### 3.2. Final Phenotype for Acute Myocardial Injury and MI

The first step in our phenotype is determining if the patient met our criteria for acute myocardial injury. Where two or more troponin measurements were available within 6 h, we examined all pairs of measurements, ensuring that at least one was at least 14 ng/L, and if any pair differed by at least 10 ng/L. In cases where two measurements were not available within 6 h, we verified if the difference between the largest and smallest values was at least 10 ng/L, and that the largest value was at least 14 ng/L. This allows us to still apply our phenotype in cases without two troponin measurements within 6 h, though the results then are more uncertain, and patients without two troponin tests will be labeled as negative. If a patient met these criteria for acute myocardial injury, we then looked for ECG report findings supporting MI, as outlined in the methods. When such findings were found the patient was labeled as having an MI, and if not with acute myocardial injury.

### 3.3. Comparison with Previous Diagnoses

In Table 4 we use the simple matching coefficient [24] to compare our phenotype with previous MI diagnoses for both cohorts. The coefficient is calculated as the number of times they agreed divided by the total number of samples. We separately consider their agreement when only an MI is considered a match, and for cases where either an MI or acute myocardial injury is considered a match.

In Table 5 and Table 6 we break down the results of Table 4, comparing the numbers and proportions of previous MI diagnoses to our phenotype labels for MI and acute myocardial injury.

## 4. Discussion

Our phenotype now enables the distinct identification of MI and acute myocardial injury in historical EMR data. All cases with two troponin measurements within two hours classified as either acute myocardial injury or MI should exhibit, at the very least, an acute myocardial injury pattern in troponin change. This implies that a considerable number of patients may lack an acute myocardial injury diagnosis, if not an MI diagnosis. This is concerning because acute myocardial injuries are associated with higher mortality levels [7]. Our phenotype can help enhance models that historically use administrative definitions and thus, produce more accurate prediction models, and in turn help to mitigate factors that increase mortality and length of stay. Patients with acute myocardial injury or MI may benefit from additional inpatient telemetry and clinical monitoring. Further investigations to diagnose underlying cardiac conditions and to tailor treatments such as medications for atherosclerotic cardiovascular disease and coronary revascularization may benefit these patients in either their index hospitalization or outpatient follow-up.

Consistent with prior research [20], our results indicate that an absolute change in troponins outperforms a relative one, in terms of agreement with previous diagnoses. In this study, we extended this comparison to multiple relative changes, as outlined in Thygesen 2012 [19], rather than the simpler single relative change tested in Mueller 2012 [20]. Despite this more nuanced approach, we still observed better performance using an absolute change. The superiority of the absolute change was evident in discrimination over the entire encounter, as well as within 6 h, justifying its application over the entire encounter. Given that fewer than 70% of patients with a diagnosed MI in either cohort had two troponin measurements within 6 h, we also provide a separate classification for these cases. Troponin values can vary due to biological and analytical variation, which can be combined into reference change values. In healthy individuals, these variations have been as significant as 86% over the course of weeks [25]. Therefore, longer interval troponin sampling introduces greater uncertainty, suggesting that a larger absolute threshold may be appropriate for certain use cases.

In Table 4, we compare our phenotype labels with previous MI diagnoses, which show a very high correspondence for diagnoses in the chart review cohort and our phenotype. However, this is due to the vast majority of the studied patients not having an MI diagnosis or two troponins available to be classified with an MI or acute myocardial injury by our phenotype, as deduced from Table 6. Nevertheless, this indicates our method is unlikely to suffer from an excessive number of false positive cases in the general inpatient population. When two troponins are available within 6 h, the CREATE diagnoses more closely match a combination of our phenotypes (acute myocardial injury or MI, 85%) rather than MI alone (70%). This is likely due to other diagnostic modalities being used in this cohort, which all underwent cardiac catheterization. The opposite is seen in the chart review cohort, though to a lesser extent (80% vs. 77%), further demonstrating our phenotype’s applicability in a general inpatient population.

Almost all patients with a diagnosed MI in either cohort had an ECG report, making them reliable sources of evidence. Since much of the ECG report is automatically populated by the MUSE system, the language is much more consistent than a standard clinical note. This consistency aids the natural language processing task, as these reports are unlikely to discuss individuals other than the patient, and negative findings are generally confined to comparisons with previous ECG report findings. However, since these reports are editable, consistency is not always guaranteed. An ECG report noting acute MI surrounded with multiple “*” is a definitive indicator, but terms like “suggests ischemia” or “possible infarction” are less certain and only meet criteria under the fourth universal definition of MI. We tested negspacy [26] for detecting negations (a python implementation of the NegEx algorithm [27]), but found it produced far more false positives than true positives for our use case. We thought it would be preferable to flag patients as positive for an MI and occasionally be incorrect, rather than miss many MIs and have treatments delayed, but be more confident that they were correctly identified.

Misclassification of myocardial infarction and myocardial injury may have several impacts. It may result in additional testing utilization and patient exposure to risk such as invasive cardiac catheterization and non-invasive cardiac imaging. It may also result in treatments that are not warranted, which are also associated with cost and risk such as procedural complications and medication side-effects. For example, percutaneous coronary intervention of a non-culprit lesion in a patient with myocardial injury misdiagnosed as myocardial infarction; or conversely, lack of appropriate secondary prevention pharmacotherapy for a patient with myocardial infarction misdiagnosed as myocardial injury.

Recently, there has been interest in SGLT2 inhibitors which are known to mitigate ischemia–reperfusion [28]. From a clinical standpoint, the effects of a baseline SGLT2 inhibitor are not known to be large enough to impact patient outcomes. While ischemia–reperfusion injury may be reduced, it does not stop myocardial injury altogether, as such, troponin thresholds and delta in our study is small, and unlikely to be impacted by the baseline use of an SGLT2 inhibitor. In clinical practice, while SGLT2 inhibitors are used in patients for blood glucose management, heart failure, and chronic kidney disease, they are not recommended for use as secondary prevention of future myocardial infarction to reduce ischemia–reperfusion injury.

The python code for parsing ECG reports is available in [23], and can be adapted for use in any EMR system or OMOP common data model [29]. The entire phenotype could theoretically be coded in Structured Query Language (SQL), making it much easier to implement in current EMR systems than advanced techniques like machine learning. However, as machine learning becomes more integrated into EMR systems, it may provide more accurate methods to extract ECG report findings. This makes our phenotype relatively easy to implement into other EMR systems, especially if they also use the MUSE ECG system and the same troponin assay. For systems that use alternative ECG systems, our NLP programs should first be validated and tailored to them if necessary. Similarly, where different high-sensitivity troponin assays are used it will be necessary to validate that our methods are still optimal.

Future work to expand our phenotype to distinguish the five types of MI defined in the fourth universal definition of MI would offer a more complete picture. This will entail broadening the types of documentation examined, such as angiography to differentiate type 1 MI from type 2, cardiac echocardiograms, and magnetic resonance imaging. The timing and documentation of procedures to distinguish procedure-related MI (type 4 and 5) from type 1 and 2 should also be explored.

### Limitations

Our phenotype effectively utilizes high-sensitivity troponins and ECG reports, but it is limited in classifying a patient with an acute myocardial injury without at least two troponin labs, or a patient with MI without an additional ECG report. This limitation may lead to the underestimating of type 3 MIs, especially when patients die before sufficient troponins or other biomarkers are collected to classify them. Additionally, ECG report findings may be susceptible to inaccuracies arising from the complexities of natural language processing or may be superseded by other evidence not included in our phenotype.

Biases in EMR documentation and data entry present an ongoing challenge to EMR phenotyping studies, as cases cannot be accurately identified if the data are not entered into the system, or if it is entered in places the algorithms do not look for it. It is important to note that this study was performed using data from one large urban city and the documentation practices in EMRs may vary across different geographic areas. Consequently, external validation of our rule-based algorithm using data from other regions and jurisdictions, including different EMR systems and different high-sensitivity troponin assays, is important to ensure our methods are generalizable. This includes different ECG systems, which are likely to format reports differently, and may pose problems for our NLP methods.

## 5. Conclusions

Our computational phenotype stands as a powerful tool for analyzing the impacts of acute myocardial injury and MI in historical EMR data. As EMR systems become increasingly prevalent, these phenotypes offer a valuable means of retrospectively examining historical data through a modern conceptual lens. Further, they hold the potential to automatically propose diagnoses for EMR problem lists, thereby enhancing the accuracy of capturing patients’ disease status. The integration of such phenotypes into healthcare systems represents a promising avenue for leveraging historical data to inform contemporary understanding and clinical decision making.

## Figures and Tables

**Table 1:** Demographics of the CREATE and Chart Review Cohorts.

*n* (%)	CREATE (*n* = 11,685 Episodes of Care)	Chart Review (*n* = 3042 Inpatient Admissions)
Age mean (SD)	63.7 (12.1)	61.1 (19.0)
Females	3130 (26.8)	1532 (50.4)
MI	7421 (63.5)	102 (3.35)
Heart Failure	1677 (14.4)	343 (11.3)
Hypertension	7989 (68.4)	1566 (51.5)
Diabetes Mellitus	3244 (27.8)	583 (19.2)
Dyslipidemia	6475 (55.4)	1030 (33.9)
Peripheral Vascular Disease	736 (6.3)	150 (4.9)
Current Smoker	2967 (25.4)	605 (19.9)

**Table 2:** Troponin coverage in the CREATE and Chart Review cohorts.

	CREATE		Chart Review		
*n* (%)	MI = Yes *n* = 7421	MI = No *n* = 4264	MI = Yes *n* = 102	MI = No *n* = 2933	MI = Maybe *n* = 7
High-Sensitivity Troponins Available					
Have at least 2 measurement	7085 (95.5)	3360 (78.8)	94 (92.2)	395 (13.5)	5 (71.4)
Have at least 2 measurements within 6 h	4753 (64.0)	1706 (40.0)	67 (65.7)	251 (8.6)	4 (57.4)
High Values (ng/L)					
At least one value > 99% URL	7194 (96.9)	2756 (64.6)	100 (98.0)	537 (18.3)	5 (71.4)
Changes Within Entire Encounter (where one measurement ≥ URL)					
if min ≤ URL: (max − min)/min > 0.5 *OR* if min > URL: (max − min)/min > 0.2	6449 (86.9)	1955 (45.8)	86 (84.3)	164 (5.6)	5 (71.4)
(max − min) ≥ 10	6696 (90.2)	1715 (40.2)	89 (87.3)	145 (4.9)	5 (71.4)
Changes Within 6 h					
if initial ≤ URL: (final − initial)/initial > 0.5 *OR* if initial > URL: (final − initial)/initial > 0.2	3487 (47.0)	423 (9.9)	48 (47.1)	56 (1.9)	3 (42.9)
|final − initial| ≥ 10	4156 (56.0)	391 (9.2)	58 (56.9)	64 (2.2)	3 (42.9)

**Table 3:** ECG report coverage in the CREATE and Chart Review cohorts.

	CREATE		Chart Review		
*n* (%)	MI = Yes *n* = 7421	MI = No *n* = 4264	MI = Yes *n* = 102	MI = No *n* = 2933	MI = Maybe *n* = 7
Have at least one report	7392 (99.6)	4122 (96.7)	101 (99.0)	1657 (56.5)	5 (71.4)
Acute MI noted *	2035 (27.4)	70 (1.6)	17 (16.7)	3 (0.1)	0 (0.0)
Infarction noted *	2744 (37.0)	260 (6.1)	24 (23.5)	32 (1.1)	0 (0.0)
Ischemia noted *	3443 (46.4)	997 (23.4)	54 (52.9)	196 (6.7)	1 (14.3)

* Note that text terms in ECG reports were only counted after exclusions as discussed in the methods.

**Table 4:** Simple matching coefficient comparing previous diagnoses to our phenotype, considering matches for MI alone and for either MI or acute myocardial injury.

	2 Troponins Within 6 h Available	2 Troponins Within 6 h Unavailable
CREATE vs. MI only	0.70	0.70
Chart * vs. MI only	0.80	0.99
CREATE vs. MI or acute myocardial injury	0.85	0.71
Chart * vs. MI or acute myocardial injury	0.77	0.98

* Chart Review MI labels of ‘maybe’ were excluded from the calculation calculations.

**Table 5:** Comparison of our phenotype for acute myocardial injury and MI with historical MI diagnoses in the CREATE cohort, based on troponin level availability.

	CREATE	CREATE MI Yes *n* (%)		CREATE MI No *n* (%)		Total
Phenotype		2 Troponin Within 6 h Yes	2 Troponin Within 6 h No	2 Troponin Within 6 h Yes	2 Troponin Within 6 h No	
Acute Myocardial Injury		1200 (25.2)	779 (29.2)	220 (12.9)	712 (27.8)	2911
Myocardial Infarction		2956 (62.2)	1368 (51.3)	171 (10.0)	265 (10.4)	4760
Neither		597 (12.6)	521 (19.5)	1315 (77.1)	1581 (61.8)	4014
Total		4753	2668	1706	2558	11,685

**Table 6:** Comparison of our phenotype for acute myocardial injury and MI with historical MI diagnoses in the Chart Review cohort, based on troponin level availability.

	Chart Review	Chart MI Yes *n* (%)		Chart MI No *n* (%)		Chart MI Maybe *n* (%)		Total
Phenotype		2 Troponin Within 6 h Yes	2 Troponin Within 6 h No	2 Troponin Within 6 h Yes	2 Troponin Within 6 h No	2 Troponin Within 6 h Yes	2 Troponin Within 6 h No	
Acute Myocardial Injury		21 (31.3)	7 (20.0)	31 (12.4)	36 (1.3)	2 (50.0)	1 (33.3)	98
Myocardial Infarction		37 (55.2)	17 (48.6)	33 (13.1)	16 (0.6)	1 (25.0)	0 (0.0)	104
Neither		9 (13.4)	11 (31.4)	187 (74.5)	2630 (98.1)	1 (25.0)	2 (66.7)	2840
Total		67	35	251	2682	4	3	3042

## Data Availability

The cohorts discussed here cannot be shared publicly, but agreements to have them analyzed can be made in cooperation with Alberta Health Services and The Centre for Health Informatics at the University of Calgary (https://cumming.ucalgary.ca/centres/centre-health-informatics/data-and-analytic-services/data-resources/requesting-ahs-data-resources, accessed on 1 January 2024).
